# A case of ileus caused by ileal endometriosis with lymph node involvement

**DOI:** 10.1016/j.ijscr.2018.11.066

**Published:** 2018-12-12

**Authors:** Ryosuke Arata, Yuji Takakura, Satoshi Ikeda, Toshiyuki Itamoto

**Affiliations:** a Department of Gastroenterological Surgery, Hiroshima Prefectural Hospital, Japan; b Department of Gastroenterological and Transplant Surgery Applied Life Sciences, Institute of Biomedical and Health Sciences, Hiroshima University, Japan

**Keywords:** CA, carbohydrate antigen, CT, computed tomography, Ileal endometriosis, Ileus, Lymph node involvement

## Abstract

**Introduction:**

Endometriosis is characterized by the presence of endometrial tissue outside the uterus. However, the presence of endometriotic lesions in extrauterine locations at the terminal ileum is unusual and a preoperative diagnosis of intestinal endometriosis has proved challenging.

**Presentation of case:**

A 49-year-old woman visited a local hospital complaining chiefly of vomiting. Computed tomography (CT) revealed an intestinal obstruction, and she was subsequently referred to and admitted to our hospital. After insertion of an ileus tube and contrast infusion, a crab-like stenosis was observed at the end of the ileum. Although its cause was unknown, we believed this stenosis to be the source of obstruction. Another CT at our hospital showed findings of obstructive ileus with a beak sign at the proximal ileum. Laparoscopic examination was performed to investigate the cause of ileus. Since the tumor at the terminal ileum was suspected to be malignant, we converted to laparotomy and performed an ileocecal excision with lymph node dissection. Based on the intraoperative pathological examination, a diagnosis of endometriosis was suspected. Another mass was found in the rectum but we ended the operation at this point. Postoperatively, the patient was referred to a gynecologist for the management of endometriosis. She was discharged on postoperative day 9 without significant complications.

**Conclusion:**

The non-specific preoperative clinical and radiological findings of ileal endometriosis make it difficult to distinguish it from other diseases. Although rare, ileal endometriosis should be considered as a differential diagnosis of intestinal obstruction in women of reproductive age.

## Highlights

• A definitive preoperative diagnosis of intestinal endometriosis is challenging.
• Intestinal endometriosis should be considered when examining premenopausal women.
• Surgery should be considered in patients presenting with intestinal obstruction.

## 1 Introduction

Endometriosis is characterized by the presence of functional endometrial tissue, consisting of glands and stroma, outside the uterus [1]. Endometriosis is found in 6–10% of women in the reproductive age group, and around 50% of affected women have associated symptoms of pelvic pain, dysmenorrhea, dyspareunia, and infertility [2]. The ovaries, uterosacral ligaments, fallopian tubes, Pouch of Douglas, and pelvic peritoneum are common sites of endometriosis, whereas the gastrointestinal (GI) tract is less frequently involved. GI tract endometriosis commonly involves the sigmoid colon and rectum; involvement of the terminal ileum is rare, comprising less than 7% of all GI tract endometriosis [3]. Furthermore, intestinal obstruction associated with endometriosis is rare, occurring in only 23% of all cases with ileal involvement [4]. Lymph node involvement in endometriosis is usually considered to be uncommon [5]. We report our experience of a patient with endometriosis of the terminal ileum that was complicated by small bowel obstruction and lymph node involvement.

This work has been reported in line with the SCARE criteria [6].

## 2 Presentation of case

**Fig. 1:**
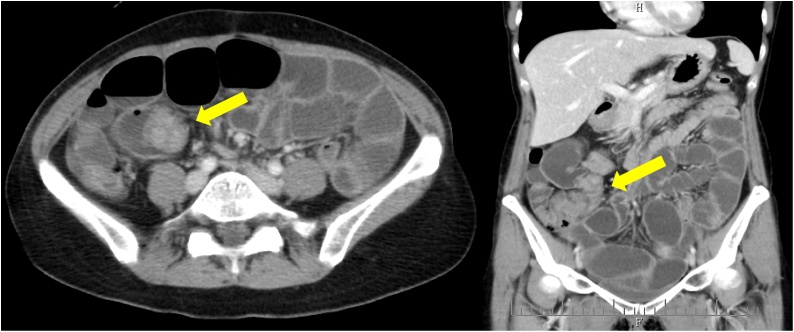
Computed tomography findings. A tumor was observed at the end of the ileum and the proximal intestinal tract was expanded, which suggested obstructive ileus (arrow).

**Fig. 2:**
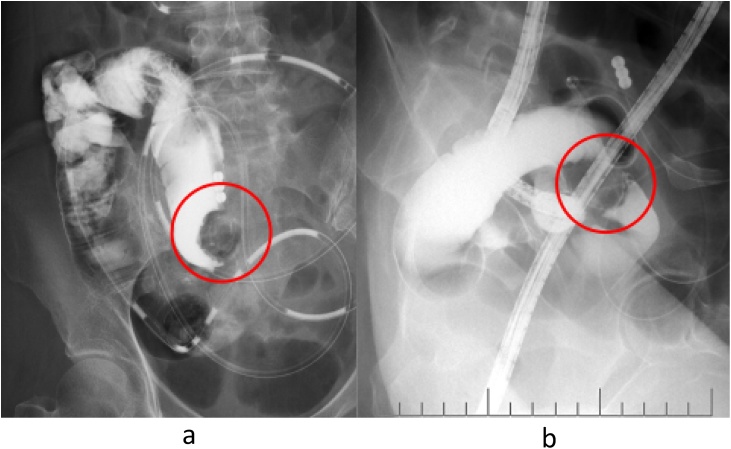
Radiographic findings. After insertion of an ileus tube and contrast infusion, a crab-like stenosis was found at the terminal ileum at the same site as the computed tomography image (a). Transanal double balloon endoscopy showed narrowing and contrast findings of 25 mm in size, 8 cm from the terminal ileum (b).

**Fig. 3:**
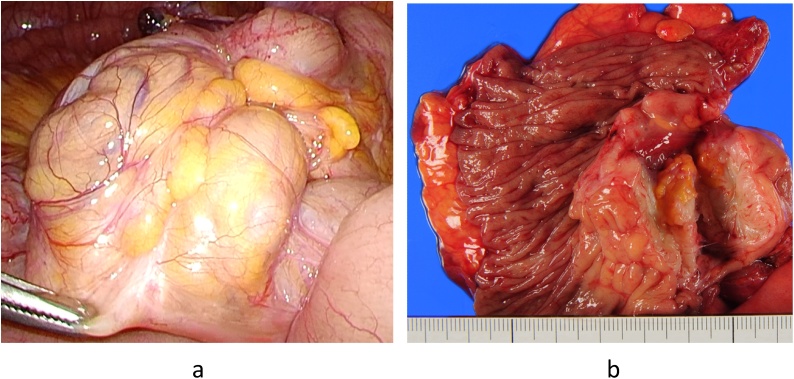
Intraoperative and postoperative findings. The macroscopic appearance was suggestive of cancer (a). The excised specimen showed thickening of the intestinal wall and stenosis of the lumen (b).

**Fig. 4:**
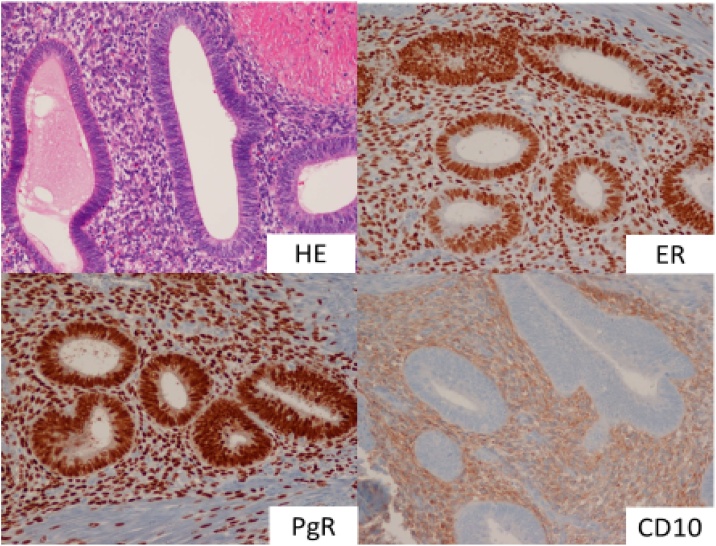
Histological and immunohistological examination of the excised specimen Endometriosis involving the ascending colon was observed (negative for estrogen and progesterone, positive for CD10).

A 44-year-old nulliparous premenopausal woman presented to a local hospital complaining chiefly of vomiting. An initial computed tomography (CT) revealed a small bowel obstruction and she was subsequently referred to our hospital. Following admission, another CT at our hospital showed a tumor at the end of the ileum and expansion of the proximal intestinal tract, indicative of ileus obstruction (Fig. 1). After insertion of an ileus tube and contrast infusion, a crab-like stenosis was found at the terminal ileum, which was the same site observed on the CT images (Fig. 2a). Transanal double balloon endoscopy showed narrowing findings of 25 mm in size, 8 cm from the terminal ileum (Fig. 2b). The cause was unknown, but the same site was judged to be the source of occlusion. Blood examination revealed a slightly increased level of carbohydrate antigen 125 (CA-125) (112 U/mL), whereas CA19-9 and carcinoembryonic antigen levels were within normal ranges (12 U/mL and 2.5 ng/mL, respectively). The patient was referred for a gynecological examination, but no findings of endometriosis or ovarian tumors were noted. Since the cause of ileus was unclear, we decided to perform a laparoscopic examination. During laparoscopy, the tumor at the terminal ileum was found. As it was suspected to be malignant, we converted to an open laparotomy and performed an ileocecal resection with lymph node dissection (Fig. 3a). Based on the intraoperative pathological examination of the tumor, a diagnosis of endometriosis was suspected. An examination of the peritoneal cavity during surgery revealed another mass in the rectum, but the operation was concluded at this point. The excised specimen showed thickening of the intestinal wall and stenosis of the lumen (Fig. 3b). Histological and immunohistological examination of the excised specimen showed endometriosis involving the ascending colon (negative for estrogen and progesterone, positive for CD10) and lymph nodes (No 201, 2 of 20) (Fig. 4). After surgery, she was referred to the gynecologist for treatment of the endometriosis. The patient was discharged without significant complications on the 9th postoperative day.

## 3 Discussion

Endometriosis usually occurs in the pelvic organs and peritoneum. However, occurrence in the rectum, colon, small intestine, kidney, ureter, appendix, external female genital organs, lymphatic nodules, or the perianal area does occur rarely. Endometriosis infiltrates the bowel in 5%–37% of cases [7], with the rectum being the most common site, followed by the sigmoid colon, appendix, ileum, and cecum.

The clinical symptoms of endometriosis include dysmenorrhea, chronic pelvic pain, dyspareunia, and infertility, but the clinical presentation is often non-specific.

Although endometriosis is not a particularly rare disease among women of childbearing age, symptoms of endometriosis are often nonspecific leading to a diagnostic dilemma. Gastrointestinal endometriosis presents with unsettling episodes of abdominal pain, abdominal distention, tenesmus, constipation, and diarrhea [8]. The preoperative diagnosis remains elusive due to clinical similarities to other causes of small bowel obstruction such as inflammatory bowel disease, infectious and ischemic colitis, and malignancy. Therefore, a definitive diagnosis can be made only after histopathological examination following surgery.

Some authors have reported that around 5% of patients with an extensive disease do not present any pain-related symptoms [9,10]. In this case, the patient did not present with typical symptoms related to endometriosis, such as dysmenorrhea, pelvic pain, or dyspareunia. Therefore, suspecting a diagnosis of ileal endometriosis in this scenario was difficult, as the patient had no features suggestive of endometriosis in her menstrual and reproductive histories.

Small bowel endometriosis should, therefore, be considered in the differential diagnosis of women of childbearing age who present with symptoms of obstruction. A high index of suspicion and prompt operative diagnosis are important, particularly when imaging tests are negative or inconclusive.

Since ileal endometriosis may act as a mass lesion and mimic malignancy by encroaching the ileal lumen, intraoperative differential diagnosis is difficult and it is possible to confuse the lesion for other tumors. Histopathological analysis is therefore required to confirm the diagnosis and assess the extent of spread [11,12]. In this patient, endometrial tissue was found in the dissected lymph nodes. Lymph node involvement in endometriosis is considered to be uncommon; however, lymph node involvement has been reported to be associated with intestinal wall infiltration by endometriosis (submucosal infiltration) [13]. However, some studies emphasize that lymph node involvement might be an underestimated event related to the minimal removal of tissues by surgeons [14]. The greatest limitation to identifying lymph node involvement is that dissection is not usually performed for benign diseases.

In a study by Noël et al. [14] of recto-sigmoid endometriosis, lymph node involvement was noted in 42.3% of cases, demonstrating that lymph node involvement in recto-sigmoid endometriosis should not be considered uncommon. A widely accepted hypothesis is that lymph node endometriosis represents the lymphatic drainage from endometriotic tissue. Endometriosis exhibits significant ability to invade the adjacent tissue with possible lymphovascular invasion, similar to true malignant tumors. While endometriosis is considered a benign disease, it is occasionally severe and progressive with a high rate of recurrence [15]. If the aim of surgery is to remove all regions with endometriosis, it may be unreasonable to remove only the visible foci when there is a high risk of associated lymph node involvement and/or recurrence [14,16]. A more conservative surgical procedure aimed at removing all visible foci followed by pharmacological therapy may be the optimal approach; however, this approach appears less effective than an extensive surgery in reducing the risk of recurrence [17]. Hence, further research may be necessary to determine the optimal surgical approach.

## 4 Conclusion

Distinguishing ileal endometriosis from other diseases based on preoperative clinical and radiological findings is difficult. Intestinal obstruction due to ileal endometriosis is a rare condition; however, it should always be considered as a differential diagnosis of intestinal obstruction in women of reproductive age. This may be of immense value in arriving at a tentative diagnosis.

## Conflict of interest

The authors have no conflicts of interest.

## Funding

This research did not receive any specific grant from funding agencies in the public, commercial, or not-for-profit sectors.

## Ethical approval

Ethical approval was not required and patient identifying knowledge was not presented in the report.

## Consent

Written informed consent has been obtained from the patient for the publication of this case report and any accompanying images.

## Author contribution

RA and YT participated in treatment of the patient, collected case details, literature search and draft the manuscript. SI participated in treatment planning of the patient. TI participated in treatment planning of the patient and helped to draft the manuscript. All authors read and approved the final manuscript.

## Registration of research studies

Not applicable.

## Guarantor

Yuji Takakura.

## Provenance and peer review

Not commissioned, externally peer-reviewed.

## References

[1] Bratu D., Chicea R., Ciprian T., Beli L., Dan S., Mihetiu A. (2016). A rare case of ileus caused by ileum endometriosis. Int. J. Surg. Case Rep..

[2] Giudice L.C. (2010). Clinical practice. Endometriosis. N. Engl. J. Med..

[3] De Ceglie A., Bilardi C., Blanchi S., Picasso M., Di Muzio M., Trimarchi A. (2008). Acute small bowel obstruction caused by endometriosis: a case report and review of the literature. World J. Gastroenterol..

[4] Macafee C.H., Greer H.L. (1960). Intestinal endometriosis. A report of 29 cases and a survey of the literature. J. Obstet. Gynaecol. Br. Emp..

[5] Gentile J.K.A., Migliore R., Kistenmacker F.J.N., Oliveira M.M., Garcia R.B., Bin F.C. (2017). Malignant transformation of abdominal wall endometriosis to clear cell carcinoma: case report. Sao Paulo Med. J..

[6] Agha R.A., Fowler A.J., Saeta A., Barai I., Rajmohan S., Orgill D.P. (2016). The SCARE statement: consensus-based surgical case report guidelines. Int. J. Surg..

[7] Namkung J., Kim S.J., Kim J.H., Kim J., Hur S.Y. (2011). Rectal endometriosis with invasion into lymph nodes. J. Obstet. Gynaecol. Res..

[8] Lin Y.H., Kuo L.J., Chuang A.Y., Cheng T.I., Hung C.F. (2006). Extrapelvic endmetriosis complicated with colonic obstruction. J. Chin. Med. Assoc..

[9] Tarjanne S., Sjöberg J., Heikinheimo O. (2009). Rectovaginal endometriosis-characteristics of operative treatment and factors predicting bowel resection. J. Minim. Invas. Gynecol..

[10] Koninckx P.R., Ussia A., Adamyan L., Wattiez A., Donnez J. (2012). Deep endometriosis: definition, diagnosis, and treatment. Fertil. Steril..

[11] Melody G.F. (1956). Endometriosis causing obstruction of the ileum. Obstet. Gynecol..

[12] Khwaja S.A., Zakaria R., Carneiro H.A., Khwaja H.A. (2012). Endometriosis: a rare cause of small bowel obstruction. BMJ Case Rep..

[13] Rossini R., Lisi G., Pesci A., Ceccaroni M., Zamboni G., Gentile I. (2018). Depth of intestinal wall infiltration and clinical presentation of deep infiltrating endometriosis: evaluation of 553 consecutive cases. J. Laparoendosc. Adv. Surg. Tech. A.

[14] Noël J.C., Chapron C., Fayt I., Anaf V. (2008). Lymph node involvement and lymphovascular invasion in deep infiltrating rectosigmoid endometriosis. Fertil. Steril..

[15] Bourdel N., Durand M., Gimbergues P., Dauplat J., Canis M. (2010). Exclusive nodal recurrence after treatment of degenerated parietal endometriosis. Fertil. Steril..

[16] Abrao M.S., Podgaec S., Dias J.A., Averbach M., Garry R., Ferraz Silva L.F. (2006). Deeply infiltrating endometriosis affecting the rectum and lymph nodes. Fertil. Steril..

[17] Cacciato Insilla A., Granai M., Gallippi G., Giusti P., Giusti S., Guadagni S. (2014). Deep endometriosis with pericolic lymph node involvement: a case report and literature review. World J. Gastroenterol..

